# Hearing Loss and Risk of Stroke and Myocardial Infarction: A Systematic Review and Meta-Analysis

**DOI:** 10.3390/jcm15020577

**Published:** 2026-01-11

**Authors:** Mengyi Wang, Yaqi Li, Juan Chen, Xin Ye, Xiang Gao

**Affiliations:** 1 Department of Nutrition and Food Hygiene, School of Public Health, Institute of Nutrition, Fudan University, Shanghai 200032, China; 2 Department of Nutrition and Food Hygiene, School of Public Health, Institute of Nutrition, Zhongshan Hospital, Fudan University, Shanghai 200032, China; 3 Institute for Global Public Policy, Fudan University, Shanghai 200433, China; 4 LSE-Fudan Research Centre for Global Public Policy, Fudan University, Shanghai 200433, China

**Keywords:** hearing loss, stroke, myocardial infarction, morbidity, meta-analysis, cerebrovascular disorders, cardiovascular disease (CVD), cardio-cerebrovascular disease (CCVD)

## Abstract

**Objective**: This systematic review and meta-analysis aims to investigate the association between hearing loss (HL) and incident cardiovascular disease (CVD), a composite of stroke and myocardial infarction (MI), and to explore the specificity of the underlying pathophysiology and the consistency of this association across key demographics and HL types. **Methods:** Adhering to PRISMA and MOOSE guidelines, we searched PubMed and Web of Science for studies published in English over the past 16 years. The analysis encompassed the spectrum of HL types. Pooled odds ratios (ORs) with 95% confidence intervals (CIs) were calculated for CVD (a composite of stroke and MI) and for each outcome separately. Extensive subgroup and sensitivity analyses were performed to assess robustness amid heterogeneity. **Results**: The analysis included 15 studies (12 cohort, 3 cross-sectional/case–control). HL was significantly associated with a high incidence of CVD (pooled OR = 1.31, 95% CI 1.05–1.65). A significant association was found for stroke (OR = 1.41, 95% CI 1.07–1.85) but not for MI (OR = 1.15, 95% CI 0.88–1.50). A consistent pattern of elevated risk was observed across all subgroups, and the primary findings remained robust in sensitivity analyses. Conclusion: Our meta-analysis indicates that HL, across its various types, is significantly associated with incident stroke, but not MI. This differential risk profile is compatible with a pathophysiology that may involve the cerebrovascular system more prominently than systemic coronary arteries. The findings highlight the potential of HL as a cost-effective indicator meriting further investigation for targeted cerebrovascular risk assessment in prevention strategies.

## 1. Introduction

Hearing loss (HL) constitutes a significant public health challenge, particularly exacerbating the disease burden among the elderly. In general, HL ranks as the third most prevalent chronic condition globally, following arthritis and hypertension. The global prevalence of HL is substantial, with approximately 466 million individuals affected in 2023 [1]. This number is likely to escalate to nearly 630 million by 2030, and could surge beyond 900 million by 2050 [2]. There is a pronounced disparity in the prevalence of HL across diverse geographical and demographic groups. The impact is particularly pronounced in less economically developed countries or regions, where the burden is disproportionately higher [3]. Therefore, the prevention of HL has emerged as a challenge spanning all age groups, warranting greater attention and prioritization.

For decades, cardiovascular disease (CVD) has been the leading cause of death globally. In 2021, 20.5 million people died from a cardiovascular condition, a figure that accounted for around one-third of all global deaths [4]. Stroke and myocardial infarction (MI) significantly contribute to this CVD burden and consistently rank among the top leading causes of death globally [5]. It is imperative to delve into a comprehensive understanding of the complications associated with MI and to develop effective preventive strategies to counteract this perilous condition [6].

Previous studies found that individuals with HL face a higher risk of stroke and other CVDs than those without HL [7,8,9,10,11,12,13,14,15,16,17]. Studies found a high prevalence of cardiovascular risk factors in people with HL, particularly its subtype of sudden sensorineural hearing loss (SSHL), possibly in part through a mechanism of microvascular disease leading to both SSHL and CVD [18,19]. HL and MI may share underlying pathophysiological pathways, including endothelial dysfunction, microvascular impairment, and chronic inflammation, which can lead to ischemic injury in both the cochlea and the myocardium. For example, microvascular injury has been proposed as a pathological mechanism in the etiology of SSHL [20]. However, previous meta-analyses regarding SSHL and incidence of stroke or MI [21,22,23] either did not include all related and recent studies to date or did not reach an agreement in terms of conclusion [21]. Although the association between HL and cardiovascular mortality is well-established [24], its relationship with the incidence of non-fatal stroke and MI—two endpoints sharing a core ischemic pathology—is not fully defined. This gap hinders our understanding of whether the underlying mechanism reflects systemic atherosclerosis or a more targeted vascular process, which our meta-analysis seeks to address. Furthermore, although MI and stroke share potential mechanistic similarities, a comprehensive meta-analysis integrating both conditions has yet to be conducted.

We thus conducted a comprehensive and updated meta-analysis to examine the occurrence of stroke and MI following HL based on studies released over the past 16 years, to calculate the aggregated and separate risks of a composite CVD of stroke and MI incidence, and to ascertain the impact of various demographic factors and types of HL on the risk of stroke and MI. The 16-year timeframe (2008–2024) was chosen to maximize methodological consistency. Older studies often lacked rigorous reporting standards (e.g., inconsistent outcome definitions, underpowered sample sizes) and are less likely to provide the granular individual data required for robust meta-analysis. Restricting the search to this period enhances the validity and reliability of our pooled results.

## 2. Methods

### 2.1. Search

We searched PubMed and Web of Science using related keywords and MeSH terms combinations (Supplementary Table S1) such as “hearing loss”, “hearing impairment”, “stroke”, “ischemic stroke” “hemorrhagic stroke”, “myocardial infarction”, “MI” “cardiovascular disease”, “CVD”, “cardio-cerebrovascular disease (CCVD)” and “CCVD” from inception to August 2025. Articles published within approximately a 16-year timeframe were considered for inclusion. Detailed tables for search terms and results are listed in Supplementary Information.

### 2.2. Study Selection

**Figure 1:**
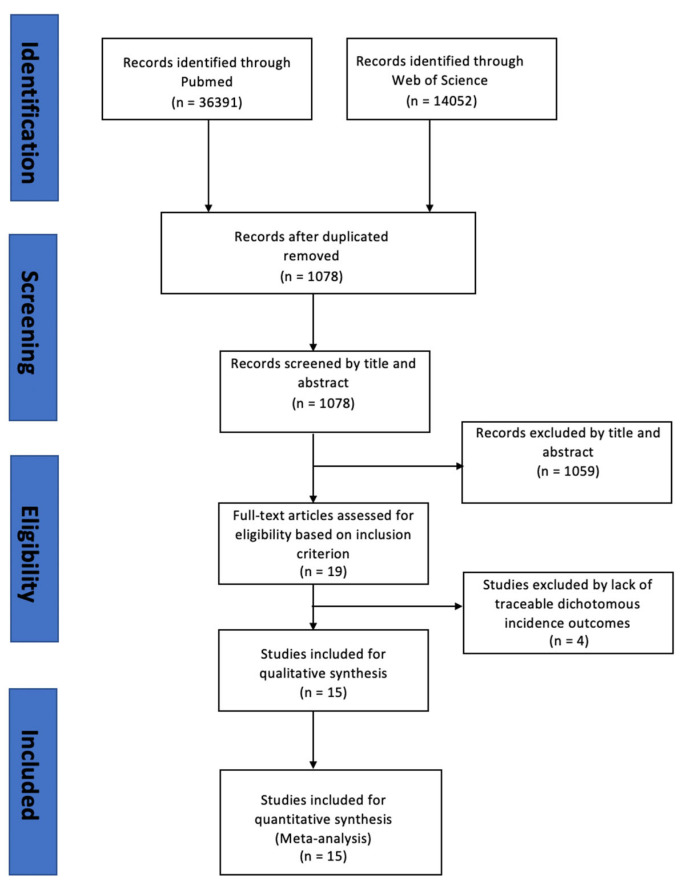
Flow diagram of the systematic review process.

We included only related papers that were published in English and within the past 16 years as of August 2025, with traceable dichotomous incidence outcomes. Due to the variability in reported effect measures across studies—such as odds ratios (ORs), hazard ratios (HRs), and relative risk (RRs)—only studies providing traceable and dichotomous incidence data were included to ensure methodological consistency. Articles that are not closely related to HL and stroke incidence were excluded. According to the flow diagram illustrated in Figure 1, after removing the duplicates, 1078 records were screened by titles and abstracts. Only articles solely examining the relationship between HL and stroke were included in the analysis. After initial screening, the remaining articles were subjected to a thorough full-text review. Studies focusing on the risk of HL following a stroke or other CVDs were excluded from the final analysis. Additional exclusion criteria were articles with unavailable incidence data after follow-up, those lacking specific definitions or categories of CVD events, studies that counted CVD and CCVD events as a single category, researches that defined CVD mortality instead of CVD incidence as outcomes, studies about dual sensory impairments without reporting separate data for hearing impairment, and articles that did not provide separate incidence data for stroke and MI. Finally, 15 studies were included in the meta-analysis according to the mentioned inclusion criteria. Pooled ORs and 95% confidence intervals (CIs) were separately calculated for studies with CVD outcomes (a composite of stroke and MI) and single outcomes (stroke only or MI only).

Complying with the Preferred Reporting Items for Systematic Reviews and Meta-Analyses (PRISMA) checklist and the Meta-analysis of Observational Studies in Epidemiology (MOOSE) recommendations [25,26], two independent reviewers conducted a thorough examination of the literature, detected and deleted duplicates based on an examination of their titles and abstracts, screened the relevance, extracted the necessary data, and evaluated the potential for bias. As Supplementary Material you can find the PRISMA checklist. 

### 2.3. Data Collection and Evaluation Process

We assessed all of the included studies carefully. Each study was reviewed by both of the authors, and then the main characteristics of the studies were recorded using Microsoft Excel version 15.40. For cohort and case–control studies, risk of bias was assessed using the Newcastle–Ottawa Scale (NOS) [27], which is recognized by the Cochrane Collaboration. Two cross-sectional studies were evaluated using the Quality Assessment Tool for Observational Cohort and Cross-Sectional Studies (QATOC) [28]. A higher score on the scale corresponds to a higher perceived quality of the research. After gathering all eligible studies, a summary table was created, and data were extracted for statistical analysis.

### 2.4. Statistical Analysis

A total of 15 studies were included in the analysis. To address the clinical heterogeneity arising from different primary outcomes across studies, multiple sets of meta-analyses were performed by grouping studies with comparable endpoints: (1) a primary analysis including all eligible studies to generate an overall pooled estimate for the composite cardiovascular disease outcome, and (2) stratified analyses for studies reporting solely on stroke or solely on MI. Primary meta-analysis included all 15 studies to generate a pooled result.

In Stata 16.0 version, random effect analysis was used to generate pooled ORs and 95% CIs. Forest plots were generated at the same time for visualization. ORs were used as the summary effect measure. This decision was made to include all eligible studies, as they reported heterogeneous effect measures (HR, OR, RR, and incident rate), so incidence outcomes were traced to generate pooled ORs. The pooled odds ratios were derived from the fundamental dichotomous outcome data (number of events and non-events, or total at-risk population) available or calculable from each study. This approach allowed all measures to be harmonized on the OR scale, maximizing data utilization and ensuring analytical consistency across the synthesis.

Under the rare disease assumption, HRs and RRs are considered comparable to ORs for pooling when outcome incidence is low, which is applicable to the general population cohorts in this study [29]. The validity of this approach was assessed in a pre-specified sensitivity analysis synthesizing HRs only (see below).

Subgroup analysis (stratified by age, geographic regions, and HL types) and meta-regression were conducted to explore sources of heterogeneity assessed using the I^2^ statistic. The variables selected for stratification were chosen based on a dual rationale: biological/clinical plausibility and practical feasibility across the included literature. Specifically, these variables represented key, well-documented, and categorically distinct population characteristics that were consistently reported in a format that permitted meaningful classification across all studies included in this synthesis [7,8,9,10,11,12,13,14,15,16,17,30,31,32,33]. Age was dichotomized (<60 vs. ≥60 years) to explore potential effect modification across major life stages. Region (Asia vs. West) was analyzed to assess the consistency of the association across distinct geographical and ethnic populations. The type of HL (sudden sensorineural, incident, or general) was examined to investigate potential mechanistic heterogeneity.

To test the robustness of the results, we conducted sensitivity analyses that excluded the following studies: (1) those with a small sample size, (2) non-cohort studies, and (3) those that used individuals with certain pre-existing diseases as controls. Additionally, to directly address methodological consistency regarding effect measures, a separate sensitivity meta-analysis was performed by synthesizing only hazard ratios (HRs), including separate analyses for composite stroke/MI outcomes (n = 8) and for stroke-only outcomes (n = 5).

Publication bias was assessed using the funnel plot and Egger’s test. The funnel plot was generated to evaluate publication bias of the association between HL and the incidence of stroke and MI as a whole for the 11 studies. Risk of bias was evaluated with the Quality Assessment Tool for Observational Cohort and Cross-Sectional Studies (QATOC).

## 3. Results

### 3.1. Study Characteristics

As illustrated in Table 1, the current meta-analysis encompasses a range of studies conducted across various geographical locations, including China (7 articles), the United States (3 articles), Korea (3 articles), Italy (1 article), Australia (1 article), and Canada (1 article). Those studies were published over a span of years, with a notable concentration in 2017 and 2018. The study designs predominantly consist of 12 cohort studies, with 1 case–control and 2 cross-sectional studies included. The follow-up periods for cohort studies range from a minimum of 2 years to a median of 14.4 years, with a total of over 10 million participants assessed. The outcomes of interest primarily focus on the association between sensorineural hearing loss of various degrees of severity and the incidence of stroke. Some studies also investigated MI. One study specifically addressed the incidence of SSHL in hemodialysis patients, highlighting a potentially higher incidence rate compared to the general population. Most of the studies reported a significantly higher risk of stroke in HL compared to those with normal hearing status.

Of the 15 included studies, 11 reported a single outcome (8 on stroke only and 3 on MI only), whereas the remaining 4 longitudinal studies reported both. Studies reporting a single outcome were pooled separately for stroke (8 studies) and MI (3 studies).

### 3.2. Synthesis of Results

#### 3.2.1. Primary Analysis

**Figure 2:**
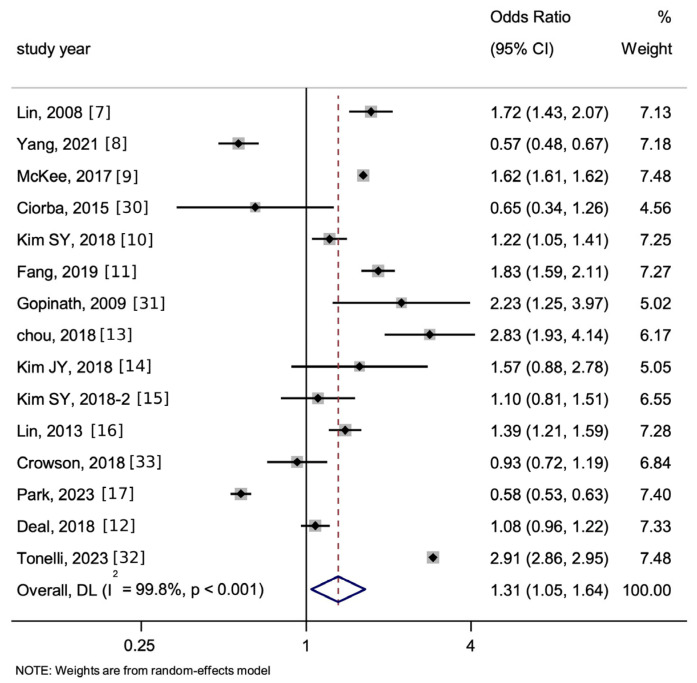
Forest plot and summary for the association between hearing loss (HL) and the incidence of stroke and myocardial infarction (MI) as a whole using a random-effects model—for all 15 studies. The notation ‘-2’ means an alternate manuscript by the same author. The dotted line represents the line of no effect (null value), and the blue diamond denotes the pooled effect estimate with its width indicating the 95% confidence interval.

As shown in Figure 2, in all 15 studies about HL predicting cardiovascular disease (CVD, a composite of stroke and MI), the overall pooled OR was 1.31 (95% [CI] 1.05–1.64, *p* < 0.05, I^2^ = 99.8%).

#### 3.2.2. Secondary Analysis

**Figure 3:**
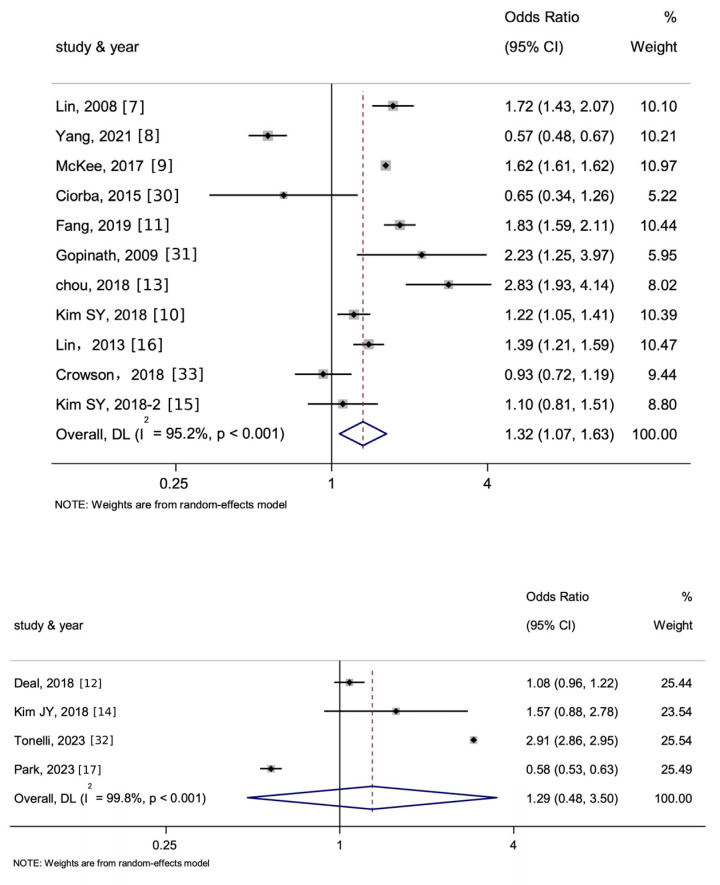
Forest plot and summary for the association between hearing loss (HL) and the incidence of stroke and myocardial infarction (MI) as a whole using a random-effects model—comparing 11 studies tracking a single outcome (**top**) and 4 studies tracking both outcomes (**bottom**). The notation ‘-2’ means an alternate manuscript by the same author. The dotted line represents the line of no effect (null value), and the blue diamond denotes the pooled effect estimate with its width indicating the 95% confidence interval.

As shown in Figure 3, among 11 studies that followed incidence of one single outcome (only stroke or only MI), the overall pooled OR of the association between HL and stroke and MI as a whole was 1.32 (95% [CI] 1.07–1.63, *p* < 0.01, I^2^ = 95.2%). Among the 4 longitudinal studies that followed both the incidence of stroke and MI, the overall pooled OR of the association between HL and stroke and MI as a whole was 1.29 (95% [CI] 0.48–3.50, *p* > 0.05, I^2^ = 99.8%).

**Figure 4:**
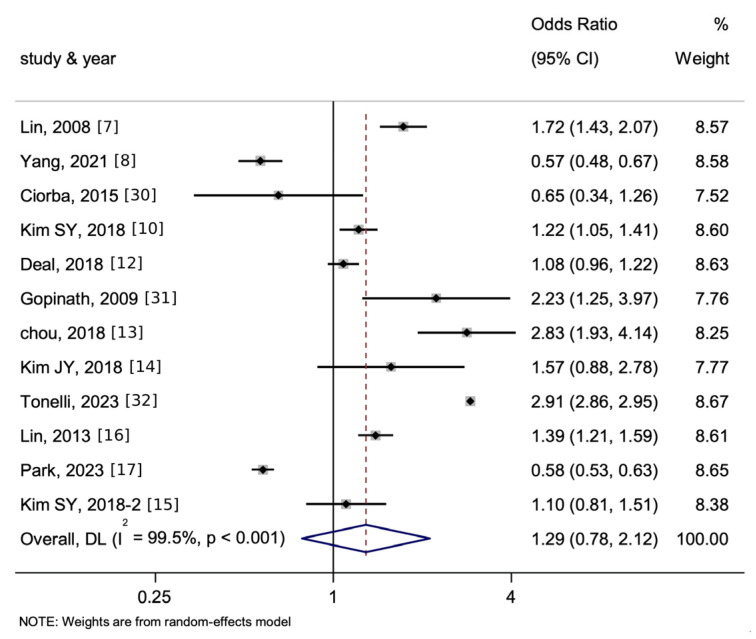
After excluding non-cohort studies, the forest plot and summary for the analysis of the association between HL and the incidence of stroke and MI as a whole. The notation ‘-2’ means an alternate manuscript by the same author. The dotted line represents the line of no effect (null value), and the blue diamond denotes the pooled effect estimate with its width indicating the 95% confidence interval.

As shown in Figure 4, for the 12 cohort studies included, the pooled OR of the association between HL and stroke and MI as a whole was 1.29 (95% [CI] = 0.78–2.12, *p* > 0.05, I^2^ = 99.5%).

**Figure 5:**
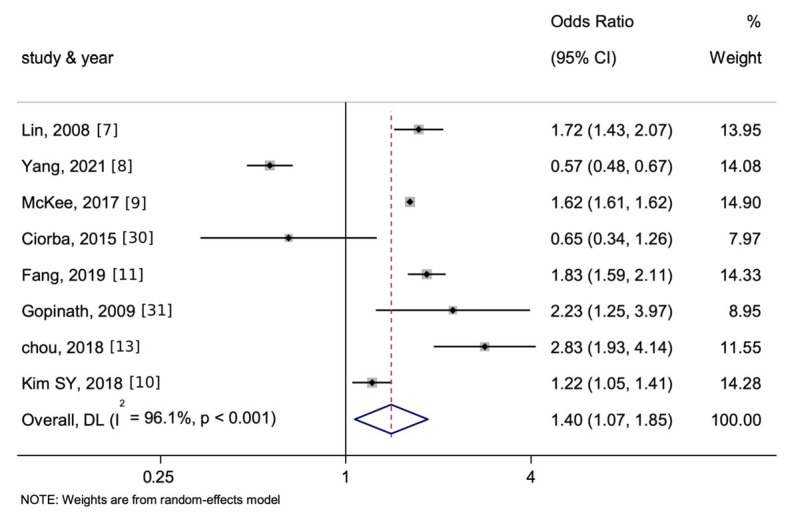

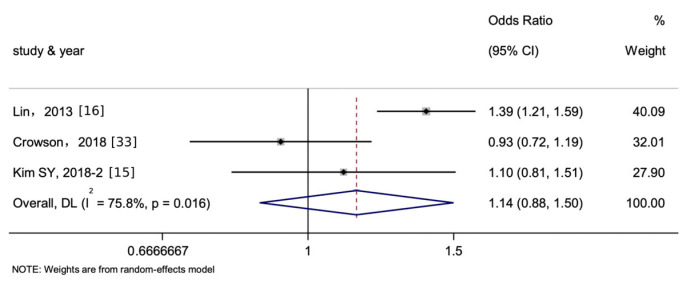
Forest plot and summary for the association between HL and the incidence of only stroke (**top**) or only MI (**bottom**) using a random-effects model—8 studies on stroke and 3 studies on MI. The notation ‘-2’ represents an alternate manuscript by the same author. The dotted line represents the line of no effect (null value), and the blue diamond denotes the pooled effect estimate with its width indicating the 95% confidence interval.

As illustrated in Figure 5, the associations of HL with stroke or MI are then listed separately. The pooled OR of the association between HL and stroke was 1.41 (95% CI = 1.07–1.85, *p* < 0.05, I^2^ = 96.1%) while that between HL and MI was 1.15 (95% CI = 0.88–1.50, *p* > 0.05, I^2^ = 75.8%).

In the meta-analysis, both stroke and MI demonstrated statistically significant associations when analyzed collectively, and only stroke demonstrated statistically significant associations with HL when analyzed separately. Individuals with HL appear to be at risk of developing cardiovascular conditions.

Specifically, in the analysis of stroke, although substantial heterogeneity remained (I^2^ = 96.1%), a large and significant effect size was observed (OR = 1.41). In contrast, the analysis of MI showed lower heterogeneity (I^2^ = 75.8%) but a smaller and non-significant effect (OR = 1.15). These results indicate a robust and significant association between HL and stroke, but not with MI, suggesting that HL may be more directly related to cerebrovascular mechanisms than to generalized atherosclerotic pathways.

#### 3.2.3. Additional Analysis

**Figure 6:**
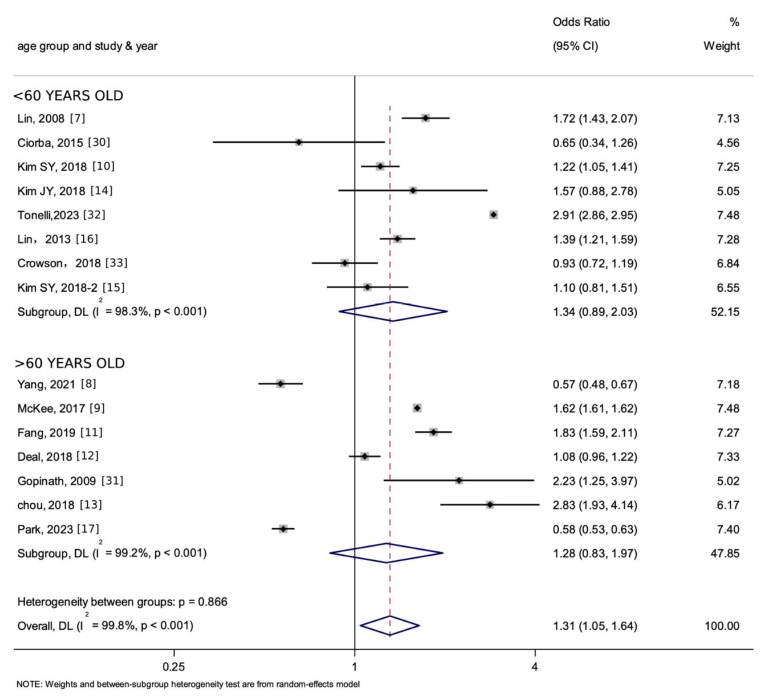
Forest plot and summary for the age subgroup analysis of the association between HL and the incidence of stroke and MI as a whole. The notation ‘-2’ represents an alternate manuscript by the same author. The dotted line represents the line of no effect (null value), and the blue diamond denotes the pooled effect estimate with its width indicating the 95% confidence interval.

***Subgroup analysis***. We conducted subgroup analysis according to age groups (Figure 6), ethnic groups (Supplementary Figure S2), and different types of HL (Supplementary Figure S3) involved. Meta-regression (Supplementary Tables S2–S4) was undertaken to assess the potential impact of the three subgroup variables on heterogeneity.

As shown in Figure 6, for individuals aged under 60, the pooled OR of the association between HL and stroke and MI as a whole was 1.34 (95% [CI] = 0.89–2.03, I^2^ = 98.3%), which suggests no significant association between HL and stroke/MI. For individuals aged above 60, the pooled OR of the association between HL and stroke and MI as a whole was 1.28 (95% [CI] = 0.83–1.97, I^2^ = 99.2%), indicating that the association was not statistically significant.

As shown in Supplementary Figure S2, for individuals living in Western countries, the pooled OR of the association between HL and stroke and MI as a whole was 1.43 (95% [CI] = 1.00–2.04, I^2^ = 99.8%). For individuals living in Asia, the pooled OR of the association between HL and stroke and MI as a whole was 1.25 (95% [CI] = 0.86–1.83, I^2^ = 99.8%).

The pooled OR was greater than 1 in all subgroups, yet the 95% CIs included the null value (1) and were statistically non-significant (*p* > 0.05). This pattern suggests a consistent positive trend across different age groups and continents, wherein HL is associated with an apparent increase in the risk of stroke/MI. However, the effect estimates within each subgroup are imprecise due to the wide CIs that cross 1.0 due to limited statistical power within each subgroup or substantial variation in the data. The possibility of no true association (OR = 1) or even a reduced risk in these specific populations cannot be ruled out. Thus, while a consistently elevated point estimate (OR > 1) across all strata suggests a uniform direction of association, the lack of statistical significance within subgroups is likely attributable to reduced statistical power after stratification and the substantial residual heterogeneity.

Similarly, as shown in Supplementary Figure S3, although the point estimates differed slightly across subgroups, the between-subgroup differences were not statistically significant. For studies defining HL as sudden HL, the pooled OR of the association between HL and stroke and MI as a whole was 1.35 (95% [CI] = 0.81–2.26, I^2^ = 99.1%, *p* > 0.05). For studies with the HL definition of incident HL, the pooled OR of the association between HL and stroke and MI as a whole was 1.06 (95% [CI] = 0.92–1.22, I^2^ = 10.2%, *p* > 0.05). For studies with general HL, which is defined as gradual HL or not specified as either sudden or incident HL, the pooled OR of the association between HL and stroke/MI as a whole was 1.49 (95% [CI] = 0.87–2.55, I^2^ = 99.2%, *p* > 0.05).

None of the three factors examined by meta-regression (all *p* > 0.05) were found to be principal contributors to the high degree of heterogeneity observed (Supplementary Tables S2–S4). In other words, the magnitude of the association did not differ significantly across populations stratified by age, geographic region, or type of hearing loss.

***Sensitivity analysis***. As shown in Supplementary Figure S4, for studies with a sample size above 3000, the pooled OR of the association between HL and stroke and MI as a whole was 1.21 (95% [CI] = 0.95–1.53, *p* > 0.05, I^2^ = 99.8%). In Supplementary Figure S5, for studies using only the general population as participants, the pooled OR of the association between HL and stroke and MI as a whole was 1.22 (95% [CI] = 0.95–1.57, *p* > 0.05, I^2^ = 99.8%).

***Sensitivity Analysis Synthesizing Hazard Ratios.*** As shown in Supplementary Figure S8, the sensitivity meta-analysis synthesizing only hazard ratios yielded a pooled HR of 4.05 (95% CI: 3.17–5.17; I^2^ = 77.2%) for the composite cardiovascular outcome (n = 8 studies). For the stroke-only outcome (n = 5 studies), the pooled HR was 5.15 (95% CI: 3.51–7.54; I^2^ = 71.7%).

The pooled HR was 4.05 (95% CI: 3.17–5.17). This estimate, while substantially higher than the primary OR-based result, reaffirms a statistically significant positive association. The divergence likely reflects the specific characteristics and potentially a higher-risk profile of the subset of studies that exclusively reported HRs.

***Publication Bias/Funnel plot.*** Visual inspection of the funnel plot was performed to evaluate publication bias. As shown in Supplementary Figure S6, most of the dots in the funnel plot cluster around the top and indicate no significant asymmetry. Egger’s test of linear regression (*p* > 0.05) also implies that there was no publication bias in this analysis (Supplementary Figure S7).

***Risk of Bias.*** Two articles were evaluated using the Quality Assessment Tool for Observational Cohort and Cross-Sectional Studies (QATOC) [28], and the rest of the articles were assessed using different sessions (case–control study or cohort study) of NOS (Newcastle–Ottawa Scale) [27] forms. Most of the articles received a bias assessment score above the 75th percentile (Supplementary Table S5).

## 4. Discussion

HL has emerged as a global health priority, affecting 466 million individuals with projections nearing 900 million by 2050, while CVD remains the leading cause of mortality worldwide. The shared vascular pathophysiology between the auditory and cardiovascular systems suggests potential mechanistic links, though existing evidence on HL-stroke/MI associations remains inconsistent due to methodological heterogeneity across studies. As accumulating evidence suggests a connection between HL and CVD, our meta-analysis of 8 studies indicates a significantly higher stroke risk among HL patients, 1.41 (95% CI = 1.07–1.85). Contrary to stroke associations, we found no conclusive evidence linking HL to independent MI risk. However, the pooled risk for the composite outcome of stroke and MI was still statistically significant (pooled OR = 1.32, 95% CI 1.07–1.63). These differential associations persisted across all subgroup strata (age, continent, and HL type), although the effects did not reach statistical significance. These findings nonetheless position HL as a potential marker for vascular health. A stronger association between HL and stroke than with MI was observed, suggesting that the link between HL and cardiovascular disease is less consistent with a predominant shared atherosclerotic pathway and raises the possibility of additional mechanisms that could particularly affect the cerebrovascular system.

High heterogeneity (>50%) was observed in all analyses, even with a large sample size of over 4 million participants. Meta-regression showed age, geographic origin (Asian vs. Western), and HL type did not substantially explain this heterogeneity, which likely stems from variations in study design, inconsistent exposure/outcome definitions, differing follow-up durations (median 2–14 years), and baseline cardiovascular risk profile differences across populations. The heterogeneity observed in our analysis was expected and directly reflects our inclusive scope.

Meta-analyses restricted to a single, narrowly-defined HL subtype (e.g., SSHL) consistently report low heterogeneity (I^2^ < 50%, even 0%), as they synthesize methodologically uniform studies. In contrast, when analyses broaden to encompass the wider spectrum of hearing loss—as ours does—high heterogeneity (I^2^ > 50% to 98%) becomes the norm, as seen in previous inclusive syntheses [21,24]. The heterogeneity in our analysis is a function of our intentionally broad inclusion criteria, which capture the real-world variation in how HL is defined and studied. We addressed this through pre-specified sensitivity and subgroup analyses to ensure the robustness of the primary findings. Therefore, our observed heterogeneity underscores the critical need for future studies to adopt uniform, objective measures (e.g., self-report vs. audiometry) to clarify these associations. The high statistical heterogeneity (I^2^ > 95%) necessitates caution in interpreting the precise magnitude of the pooled effect estimate. It indicates that the true effect may vary considerably across different populations and study settings. However, the consistency in the direction of effect (all point estimates > 1) across all primary and sensitivity analyses, coupled with the statistically stable finding for stroke, suggests that the core association is reliable despite this variability.

Our meta-analysis intentionally synthesized evidence across the entire spectrum of HL—from sudden to age-related—to provide a generalizable, real-world estimate of its association with cardiovascular outcomes. The substantial statistical heterogeneity (I^2^ > 95%) we observed is a direct and expected consequence of this inclusive clinical approach, which captures the true diversity in HL definitions, populations, and settings. This stands in contrast to previous syntheses that, by focusing on a single HL subtype (e.g., SSHL), achieved low heterogeneity but at the cost of narrow clinical applicability. Our comprehensive scope is the study’s key strength, and the heterogeneity it introduces is not a bias, but a feature we rigorously managed. Critically, despite this expected statistical diversity, our core finding was robust: the association between HL and stroke remained significant and stable across all sensitivity analyses. Most importantly, we observed a consistent, positive direction of effect across every single subgroup. This powerful consistency in the face of clinical variability underscores the fundamental robustness of the HL-stroke link and its relevance to a broad patient population.

The markedly divergent findings for stroke and MI are central to our interpretation. The analysis of stroke, encompassing millions of participants, demonstrated a robust and significant association (OR = 1.41, 95% CI 1.07–1.85). In contrast, while the MI analysis also drew from a large population base, it accrued a much smaller number of clinical events (approximately 1328). This lower event rate limits our power to detect a very weak association. However, the resulting odds ratio of 1.15, with a narrow CI spanning a clinically negligible range (0.88–1.50), provides compelling evidence against any strong or meaningful association. While this analysis cannot definitively exclude a potential minor, system-wide vascular contribution—which might be undetected due to the more limited number of MI events—the marked disparity in effect sizes between stroke and MI is less supportive of a dominant shared atherosclerotic mechanism. This pattern may indicate heterogeneity in the underlying mechanisms and raises the possibility that factors affecting cerebrovascular integrity are particularly involved.

This finding that HL is associated with CVD or stroke aligns with the conclusions of previous meta-analyses [21,22,23], and the collective results from a considerable body of articles in this study support the association between HL and stroke/MI as a whole. The relationship between high-frequency HL and CVD was noted in an earlier meta-analysis [22] but was not available in this study. Previous meta-analyses [23] mentioned that individuals with SSHL have a significantly higher risk of stroke in comparison to individuals with age-related HL. However, in this study, we found no statistically significant risk difference between different HL types. A previous meta-analysis mentioned that individuals above 50 years-old and with HL have a significantly higher risk of stroke in comparison to other age groups [8], but in our findings, there is no risk difference across age groups. Prior research, such as the meta-analysis on mortality [24], linked HL to the terminal outcome and concluded that the risk of all-cause mortality was slightly higher in studies from Asia compared to other continents. In contrast, our findings regarding the association with non-fatal stroke and MI implicate HL in the disease pathogenesis at a different and potentially more modifiable stage, and we found that, despite non-significant subgroup differences, an elevated risk may exist across all regions. HL subtypes and MI were not the focus of previous meta-analyses.

The association between HL and CVD has often been attributed to shared systemic pathways, including endothelial dysfunction and microvascular impairment [34,35], as well as generalized arteriosclerotic processes that may compromise blood supply to the metabolically active cochlea [36,37,38]. However, our findings are less supportive of the predominance of this generalized model. The significant association with stroke, in contrast to the null association with MI, suggests the need to explore additional pathways that may particularly involve the cerebrovascular system.

This specificity may be explained by the unique vulnerability of the cochlear and central auditory vascular supply to the same small vessel disease and hypoperfusion that underlie cerebrovascular pathology. Furthermore, the established link between HL and cognitive decline—a condition strongly driven by cerebrovascular damage—provides indirect support for a shared cerebrovascular etiology. While hypertension remains a common risk factor for both HL and CVD, potentially operating through mechanisms such as blood-labyrinth barrier disruption [39,40], our results suggest its impact may be particularly consequential within the cerebrovascular-auditory axis.

This analysis incorporated an unprecedented number of high-quality studies about HL-CVD incidence within recent decades and comprehensively included a sizable number of cohort studies (the largest number of cohort studies compared to previous meta-analyses) characterized by robust quality, substantial sample size, and a follow-up period of at least two years. Several closely related cross-sectional studies were also included to generate a comprehensive pooled OR. Instead of mainly focusing on SSHL, different categories of HL were noted, classified, and analyzed separately in this meta-analysis, which has not been done in previous studies. Also, in this study, stroke and MI were distinctively investigated, with their shared underlying mechanisms. Risk of bias was evaluated via a more detailed approach under two representative systems.

This study has several limitations that should be acknowledged. First, the sensitivity analysis restricting inclusion to higher-quality studies (reduced sample size) might raise concerns about generalizability and statistical power. However, in this restricted analysis, the point estimate for the association between HL and stroke was not only preserved but strengthened (OR = 1.52) and remained statistically precise. This pattern supports the finding that the observed link is not an artifact of lower-quality studies and underscores the robustness of the association in the highest-grade evidence. Large-scale prospective studies will be able to quantify the effect size more precisely. Second, the evidence quality is moderate due to the inclusion of retrospective cohort and non-cohort studies. Third, differences in epidemiological data (e.g., disease coding systems) across national databases may act as confounders. Additionally, limited stroke subtype data restricts understanding HL’s contributions to different stroke categories, and unadjusted results in some studies call for further analysis accounting for confounders like smoking, BMI, diet, and occupational/environmental exposures. Fourth, the use of a composite stroke outcome (combining ischemic and hemorrhagic subtypes) may obscure clinically important differences, as these events have distinct pathophysiological mechanisms. Our analysis aimed to provide an overall estimate of the HL–cerebrovascular risk link; future studies with detailed subtype data are needed. Fifth, as with any meta-analysis of observational studies, residual confounding by unmeasured or imperfectly adjusted factors (such as detailed smoking history, blood pressure control, specific medication use, or environmental exposures) could influence the observed associations and remains an inherent limitation. Regarding the synthesis of effect measures, our primary analysis pooled ORs from studies reporting HRs, RRs, and ORs to ensure inclusiveness. A sensitivity analysis restricted to HRs yielded higher point estimates, highlighting that the choice of effect measure and the subset of studies can influence the result. Therefore, while the primary OR-based analysis provides the most comprehensive summary, the reported association should be interpreted as a measure of strength rather than a precise risk ratio, and the estimates from the HR-only sensitivity analysis should be considered as reflecting a specific subset of the evidence. Furthermore, we could not perform subgroup analysis by sex due to a lack of sex-stratified data in the primary studies. Similarly, subgroup analyses based on specific comorbidities (e.g., hypertension, diabetes) were not feasible due to inconsistent definitions and reporting across the primary studies, which limits our ability to examine these potential effect modifiers. Also, the analysis of MI had limited power to detect a weak association due to a lower number of endpoint events; therefore, a potential minor, system-wide vascular contribution cannot be entirely ruled out.

Our findings position HL, a highly prevalent and often readily identifiable condition, as a potential novel correlate of CVD risk. This suggests that the utility of routine audiological assessment within primary care and cardiovascular risk stratification protocols warrants prospective evaluation to determine if it can help identify asymptomatic individuals who may benefit from intensified screening and management of modifiable stroke risk factors (e.g., hypertension, atrial fibrillation). From a public health perspective, investigating whether HL could serve as a warning signal represents a pragmatic research direction for potentially shifting stroke prevention towards an earlier stage. Given the global burden of both conditions, assessing the population-level impact of such an approach warrants further study.

To translate this evidence into clinical practice, future research needs to first address the heterogeneity in the current literature by prospectively collecting standardized, objective audiometric data alongside detailed cardiovascular phenotyping. Crucially, studies should incorporate neuroimaging markers of cerebral small vessel disease (e.g., white matter hyperintensities, silent infarcts) to explore the proposed cerebrovascular mechanism. Ultimately, a necessary step will be to design and implement interventional trials to determine whether identifying and addressing HL can lead to improved cardiovascular risk profiles or a tangible reduction in stroke incidence.

## 5. Conclusions

This meta-analysis suggests that HL is a significant and specific risk marker for incident stroke, but not for MI. The robustness of the primary association for stroke was confirmed through sensitivity analyses, despite substantial heterogeneity inherent to synthesizing diverse HL types and study populations. This distinct risk profile is compatible with, but does not prove, the hypothesis that the underlying pathophysiology may be more closely linked to cerebrovascular integrity than to a generalized atherosclerotic process, but further longitudinal and interventional studies are needed. Consequently, HL assessment—across its various presentations—might be considered a potential indicator for future research to explore whether it can help identify individuals who may benefit from targeted cerebrovascular risk evaluation. Future research should prioritize prospective designs with objective hearing measures and incorporate neuroimaging biomarkers to help clarify the causal pathway and explore potential interventions.

## Figures and Tables

**Table 1:** Summarization of the studies included in the meta-analysis.

Author, Year	Country	Study Design	Study Population	HL Definition	Stroke Definition	MI Definition	Follow up, Years	Results
**Lin, 2008** [7]	China	Cohort	7736	SSHL (ICD-9-CM: 388.2)	Any stroke (ICD-9-CM: 430–438)	N/A	5.0	Stroke HR: 1.64 (1.31–2.07)
**Yang, 2021** [8]	China	Cohort	13,880	PTA > 25 dB (Severe: >60 dB)	Incident stroke (Clinical & CT/MRI confirmed)	N/A	5.0	Severe HL vs. Normal: Stroke RR: 1.51 (1.03–2.20)
**McKee, 2017** [9]	USA	Cross-sectional Study	11,173	Self-reported “a little trouble” or worse	Self-reported diagnosis	N/A	N/A	Stroke OR: 1.39 (1.12–1.66)
**Ciorba, 2015** [30]	Italy	Retrospective Cohort	353,622	SSHL (ICD-9-CM: 388.2)	Ischemic stroke (ICD-9-CM codes)	N/A	Mean: 6.0	Ischemic Stroke HR: N.S.
**Kim SY, 2018** [10]	Korea	Longitudinal Cohort	24,720	SSHL (ICD-10: H91.2, audiometry & steroid treated)	Ischemic/Hemorrhagic stroke (ICD-10 codes)	N/A	Mean: 4.8	Ischemic Stroke HR: 1.22 (1.05–1.43)
**Fang, 2019** [11]	China	Cross-sectional Study	19,238	PTA > 25 dB (Severe: >60 dB)	Definite stroke (ICD-10 codes & physician confirmed)	N/A	N/A	Severe HL vs. Normal: Stroke OR: 1.76 (1.30–2.38) (speech frequency)
**Deal, 2018** [12]	USA	Retrospective Cohort	203,994	Incident HL (2 claims within 2 years)	Incident stroke (CMS CCW algorithms)	Incident MI (CMS CCW algorithms)	10.0	Attributable Risk/100 persons: Stroke: 2.69 (1.03–4.35); MI: 1.05 (0.04–2.07)
**Gopinath, 2009** [31]	Australia	Cohort	1394	PTA > 25 dB in better ear	Incident stroke (MONICA criteria, neuroimaging confirmed)	N/A	5.0	Stroke OR: 1.14 (0.59–2.23)
**Chou, 2018** [13]	China	Cohort	1956	SSHL (ICD-9-CM codes)	Hemorrhagic & Ischemic stroke (ICD-9-CM codes)	N/A	11.0	Competing Risk aHR: Hemorrhagic: 4.08 (1.93–8.61); Ischemic: 2.34 (1.45–3.78)
**Kim JY, 2018** [14]	Korea	Retrospective Cohort	770	SSHL (KCD codes, PTA & steroid treated)	Stroke (KCD codes: I60–I63)	AMI (KCD code: I21)	11.0	CCVD HR: 2.18 (1.20–3.96); Stroke HR: 2.02 (1.16–3.51); MI HR: 1.18 (0.25–5.50).
**Tonelli, 2023** [32]	Canada	Retrospective Cohort	4,724,646	HL (Algorithmically identified via healthcare claims using ICD codes).	Stroke/TIA (First occurrence)	MI (First occurrence)	Median: 14.4	Stroke/TIA HR: 1.37 (1.35–1.39); MI HR: 1.10 (1.07–1.13)
**Kim SY,** **2018-2** [15]	Korea	Longitudinal Cohort	22,099	SSHL (ICD-10 codes, PTA & steroid treated)	N/A	MI (ICD-10: I21)	11.0	MI HR: 1.39 (1.00–1.93)
**Lin, 2013** [16]	China	Cohort	88,811	SSHL (ICD-9: 388.2)	N/A	MI (ICD-9: 410)	Min: 3.0	MI HR: 1.25 (1.09–1.44)
**Crowson, 2018** [33]	USA	Case–control	21,213	ISSHL (ICD-9 codes, steroid prescription within 7 days)	N/A	AMI (ICD-9 codes)	N/A	AMI HR: 0.86 (0.67–1.10)
**Park, 2023** [17]	Korea	Cohort	38,426	SSHL (ICD-10: H912)	Stroke (ICD-10: I60–I64)	AMI (ICD-10: I21)	17.0	CCVD HR: 1.17 (1.11–1.24); Stroke HR: 1.17 (1.10–1.25)

Abbreviations: HL, hearing loss; SSHL, sudden sensorineural hearing loss; ISSHL, idiopathic sudden sensorineural hearing loss; MI, myocardial infarction; CCVD, cardio-cerebrovascular disease; OR, odds ratio; HR, hazard ratio; RR, risk ratio; ICD, International Classification of Diseases; CM, clinical modification; KCD, Korean Classification of Diseases; N.S., Not Significant; CMS CCW, the Centers for Medicare and Medicaid Services Chronic Conditions Data Warehouse; MONICA, Multinational MONItoring of trends and determinants in CArdiovascular disease; aHR, adjusted hazard ratio; PTA, pure-tone average (dB); CT, computed tomography; MRI, magnetic resonance imaging; TIA, transient ischemic attack; AMI, acute myocardial infarction; QATOC, Quality Assessment Tool for Observational Cohort and Cross-Sectional Studies. Note: The notation ‘-2’ represents an alternate manuscript by the same author. For McKee et al. (2017) [9], the sample size (N = 11,173) refers to the final analytical cohort with complete data for all variables. The initial pooled NHIS sample was 53,111. Formatting note: Bold font is used in the table header row and the first column (“Author, Year”) to provide clear visual distinction from the data cells and to aid in horizontal tracking across rows.

## Data Availability

No new data were generated or analyzed in support of this research.

## Supplementary Materials

The following supporting information can be downloaded at: https://www.mdpi.com/article/10.3390/jcm15020577/s1, Supplementary Table S1: PubMed search terms and results; Supplementary Table S2: Meta-regression results for subgroup (age); Supplementary Table S3: Meta-regression results for subgroup (geographic regions); Supplementary Table S4: Meta-regression results for subgroup (types of hearing loss); Supplementary Table S5: Overview of Risk of Bias Evaluation Scores; Supplementary Figure S1: Categories of studies with comparable endpoints and flow diagram of analysis process; Supplementary Figure S2: Forest plot and summary for the continent subgroup analysis of association between HL and the incidence of stroke and MI as a whole; Supplementary Figure S3: Forest plot and summary for the HL type subgroup analysis of association between HL and the incidence of stroke and MI as a whole; Supplementary Figure S4: After including studies with sample size above 3000, forest plot and summary for the sensitivity analysis of association between HL and the incidence of stroke and MI as a whole; Supplementary Figure S5: Forest plot and summary of the sensitivity analysis for the association between hearing loss and the incidence of stroke and MI, excluding studies not based on the general population; Supplementary Figure S6: Among 11 studies inspecting 1 single CVD outcome (stroke only or MI only). Funnel plot for the association between HL and the incidence of stroke and MI as a whole; Supplementary Figure S7: Egger’s regression test for the association between HL and the incidence of stroke and MI as a whole. Supplementary Figure S8: Forest plots of sensitivity meta-analyses synthesizing hazard ratios (HRs) only. (left) Composite outcome of stroke or myocardial infarction (MI) (n = 8 studies). (right) Stroke outcome only (n = 5 studies). PRISMA checklist: PRISMA 2020 for Abstract Checklist.
