# Supplementary material for: Hearing Loss and Risk of Stroke and Myocardial Infarction: A Systematic Review and Meta-Analysis
Source: J Clin Med. 2026 Jan 11;15(2):577. doi: 10.3390/jcm15020577 (PMC12841837; doi:10.3390/jcm15020577)

Supplementary Information

Supplementary Table S1. PubMed search terms and results.

PubMed	
**Search Terms**	**Number of Results**
(Hearing Loss) AND (Stroke)	996
(Hearing Loss) AND (Myocardial Infarction)	101
(Hearing Loss) AND (Acute Myocardial Infarction)	27
(Hearing Loss) AND (MI)	2485
(Hearing Loss) AND (Cardiovascular Diseases)	4956
(Hearing Loss) AND (Cardiovascular Disease)	5217
(Sensorineural Hearing Loss) AND (Cardiovascular Disease)	2021
(Sensorineural Hearing Loss) AND (Cerebrovascular Disease)	659
(Hearing Loss) AND (CVD)	0
(Hearing Loss) AND (CCVD)	3
(Hearing Loss) AND cardiovascular	2229
(Hearing Loss) AND (ischemic stroke)	128
(Hearing Loss) AND (hemorrhagic stroke)	12
(Hearing Impairment) AND (Stroke)	1243
(Hearing Impairment) AND (Myocardial Infarction)	109
(Hearing Impairment) AND (Acute Myocardial Infarction)	31

(Hearing Impairment) AND (MI)	2647
(Hearing Impairment) AND (Cardiovascular Diseases)	5308
(Hearing Impairment) AND (Cardiovascular Disease)	5597
(Hearing Impairment) AND (CVD)	47
(Hearing Impairment) AND (CCVD)	3
(Hearing Impairment) AND cardiovascular	2408
(Hearing Impairment) AND (ischemic stroke)	151
(Hearing Impairment) AND (hemorrhagic stroke)	13
**Web of Science**	
**Search Terms**	**Number of Results**
"Hearing Loss" AND "Stroke"	2153
"Hearing Loss" AND "Myocardial Infarction"	424
"Hearing Loss" AND "Acute Myocardial Infarction"	50
"Hearing Loss" AND "MI"	78
"Hearing Loss" AND "Cardiovascular Diseases"	668
"Hearing Loss" AND "Cardiovascular Disease"	739
"Hearing Loss" AND "Cerebrovascular Disease"	158
"Sensorineural Hearing Loss" AND "Cardiovascular Disease"	112

“Sensorineural Hearing Loss” AND “Cerebrovascular Disease”	44
“Hearing Loss” AND “CVD”	67
“Hearing Loss” AND “CCVD”	3
“Hearing Loss” AND “cardiovascular”	6281
“Hearing Loss” AND “ischemic stroke”	169
“Hearing Loss” AND “hemorrhagic stroke”	48
“Hearing Impairment” AND “Stroke”	737
“Hearing Impairment” AND “Myocardial Infarction”	97
“Hearing Impairment” AND “Acute Myocardial Infarction”	14
“Hearing Impairment” AND “MI”	20
“Hearing Impairment” AND “Cardiovascular Diseases”	368
“Hearing Impairment” AND “Cardiovascular Disease”	243
“Hearing Impairment” AND “Cerebrovascular Disease”	50
“Hearing Impairment” AND “CVD”	34
“Hearing Impairment” AND “CCVD”	0
“Hearing Impairment” AND “cardiovascular”	1461
“Hearing Impairment” AND “ischemic stroke”	28
“Hearing Impairment” AND “hemorrhagic stroke”	6

Supplementary Table S2. Meta-regression results for subgroup (age).

Meta-regression	Number of obs = 15
REML estimate of between-study variance	tau2 = .268
% residual variation due to heterogeneity	I-squared_res = 98.86%
Proportion of between-study variance explained	Adj R-squared = -7.20%
With Knapp-Hartung modification	

logor	Coef.	Std. Err.	t	P>|t|	[95% Conf.	Interval]
older	-.060245	.2789815	-0.22	0.832	-.6629478	.5424578
_cons	.2991107	.1923374	1.56	0.144	-.1164091	.7146305

Supplementary Table S3. Meta-regression results for subgroup (geographic regions).

Meta-regression	Number of obs = 15
REML estimate of between-study variance	tau2 = .2643
% residual variation due to heterogeneity	I-squared_res = 99.80%
Proportion of between-study variance explained	Adj R-squared = -5.71%
With Knapp-Hartung modification	

logor	Coef.	Std. Err.	t	P>|t|	[95% Conf.	Interval]
western	.1217313	.2840657	0.43	0.675	-.4919554	.735418
_cons	.2229947	.1773735	1.26	0.231	-.1601975	.6061869

Supplementary Table S4. Meta-regression results for subgroup (types of hearing loss).

Meta-regression	Number of obs = 16
REML estimate of between-study variance	tau2 = .2466
% residual variation due to heterogeneity	I-squared_res = 98.87%
Proportion of between-study variance explained	Adj R-squared = -4.17%
With Knapp-Hartung modification	

logor	Coef.	Std. Err.	t	P>|t|	[95% Conf.	Interval]
general	.1847364	.2676179	0.69	0.501	-.389247	.7587198
_cons	.2074083	.1671828	1.24	0.235	-.1511632	.5659798

Supplementary Table S5. Overview of Risk of Bias Evaluation Scores. Maximum scores for cohort, case–control, and cross-sectional studies are 13, 10, or 14, respectively. CS stands for cross-sectional study. CC stands for case–control study.

#	Author (Year)	Selection maximum score = 4 (CC) or 6 (cohort)	Comparability (maximum score = 2)	Exposure/ Outcome maximum score = 4 (CC) or 5 (cohort)	Total maximum score = 10 (CC) or 13(cohort)
1	Lin, 2008	3	2	1	6
2	Yang, 2021	6	2	2	10
3	McKee, 2017 (CS)	NA	NA	NA	8
4	Ciorba, 2015	4	0	2	6
5	Kim SY, 2018	5	2	2	9
6	Fang, 2019 (CS)	NA	NA	NA	8
7	Deal, 2018	3	3	2	8
8	Gopinath, 2009	5	2	2	9
9	Chou, 2018	4	2	2	8
10	Kim JY, 2017	5	2	2	9
11	Tonelli, 2023	4	2	2	8
12	Kim SY, 2018-2	5	2	2	9
13	Lin, 2013	3	2	2	7
14	Crowson， 2018 (CC)	3	2	1	6
15	Park, 2023	4	2	2	8

Supplementary Figure S1. Categories of studies with comparable endpoints and flow diagram of analysis process.

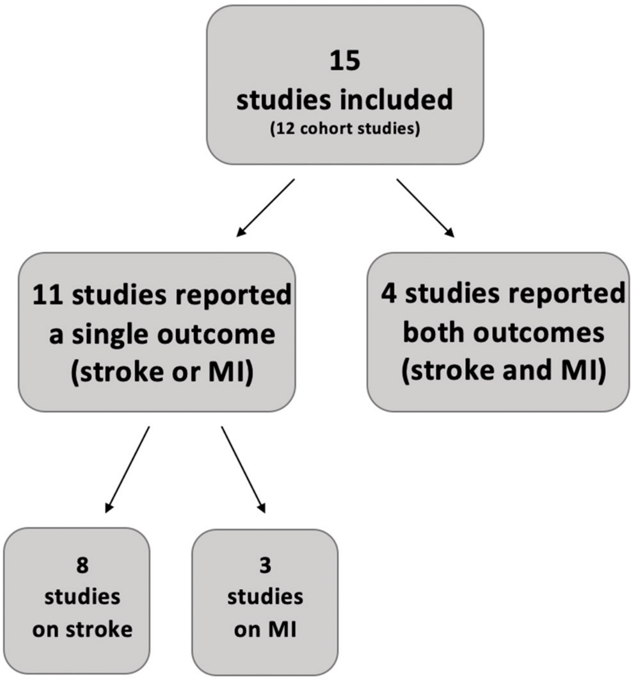

Supplementary Figure S2. Forest plot and summary for the continent subgroup analysis of the association between HL and the incidence of stroke and MI as a whole. The notation '-2' is an alternate manuscript by the same author.

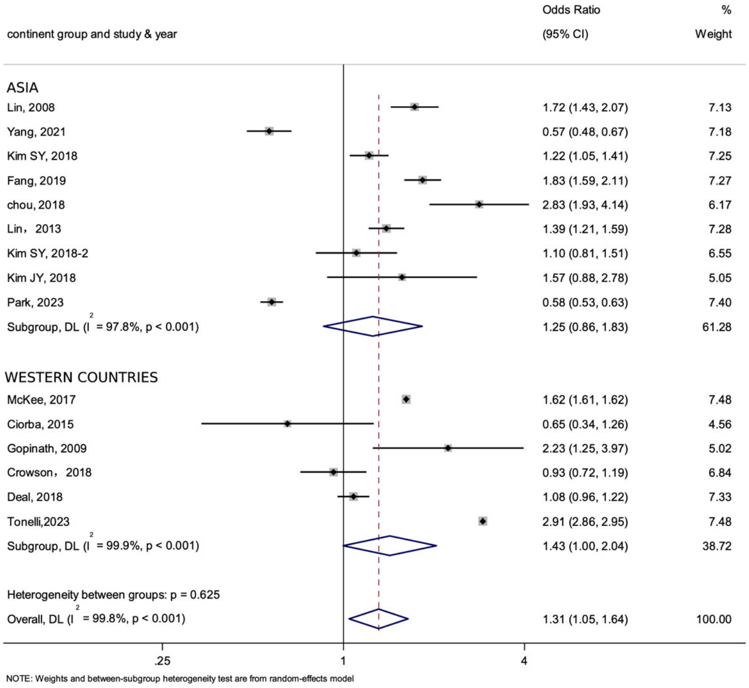

Supplementary Figure S3. Forest plot and summary for the HL type subgroup analysis of the association between HL and the incidence of stroke and MI as a whole. The notation '-2' is an alternate manuscript by the same author.

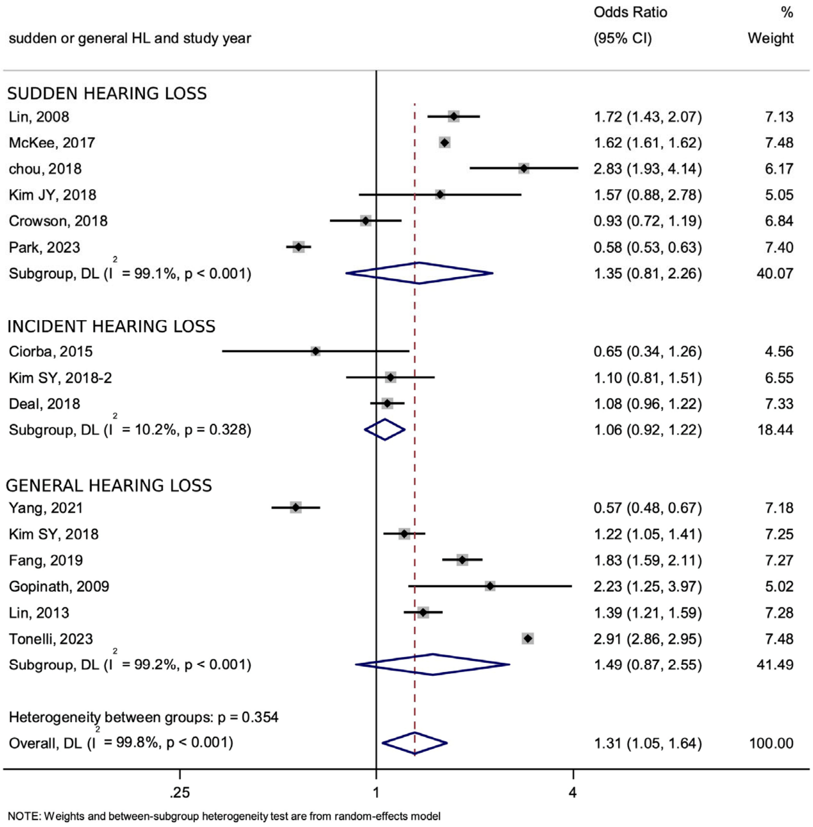

Supplementary Figure S4. After including studies with a sample size above 3000, the forest plot and summary for the sensitivity analysis of the association between HL and the incidence of stroke and MI as a whole are shown.

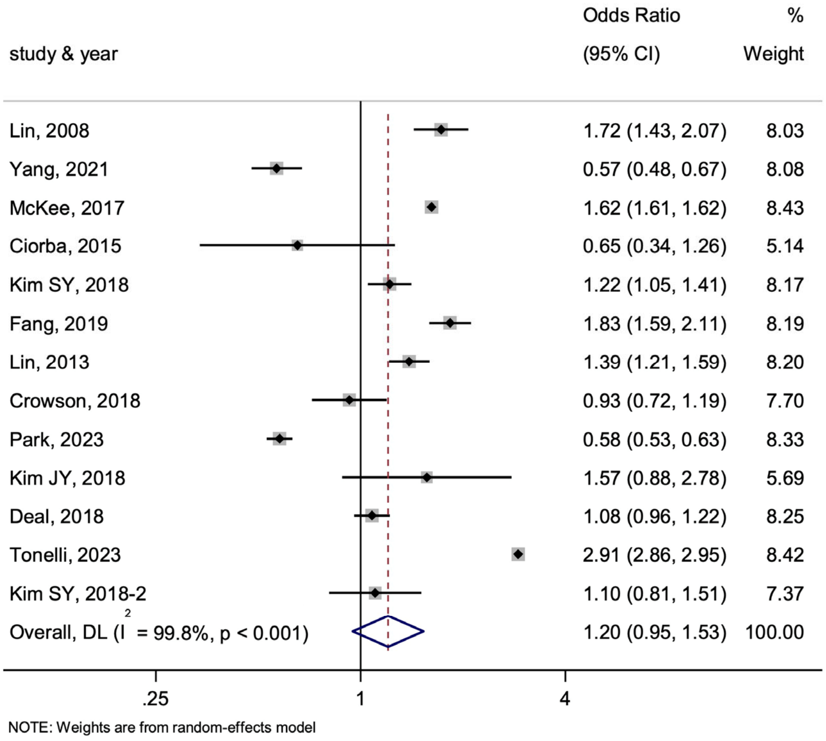

Supplementary Figure S5. Forest plot and summary of the sensitivity analysis for the association between hearing loss and the incidence of stroke and MI, excluding studies not based on the general population.

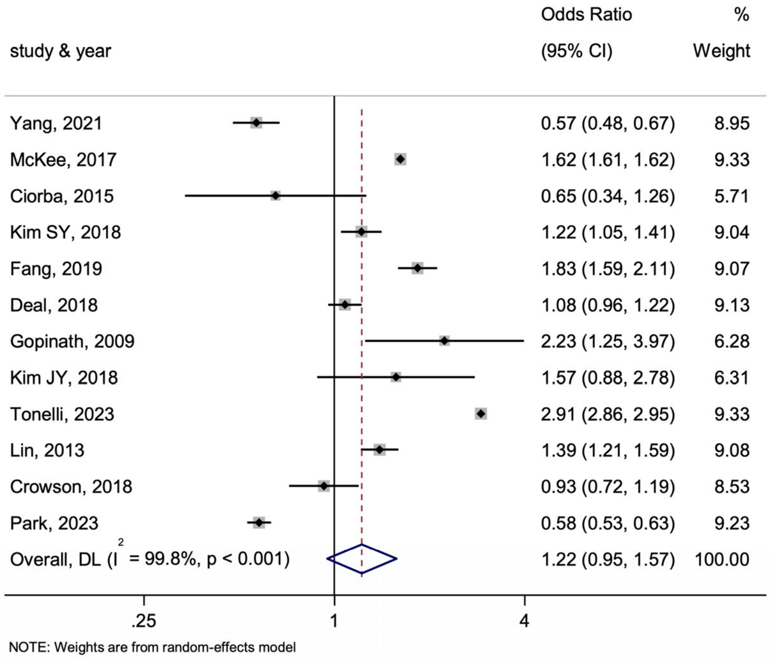

Supplementary Figure S6. The 11 studies inspecting 1 single CVD outcome (stroke only or MI only). Funnel plot for the association between HL and the incidence of stroke and MI as a whole.

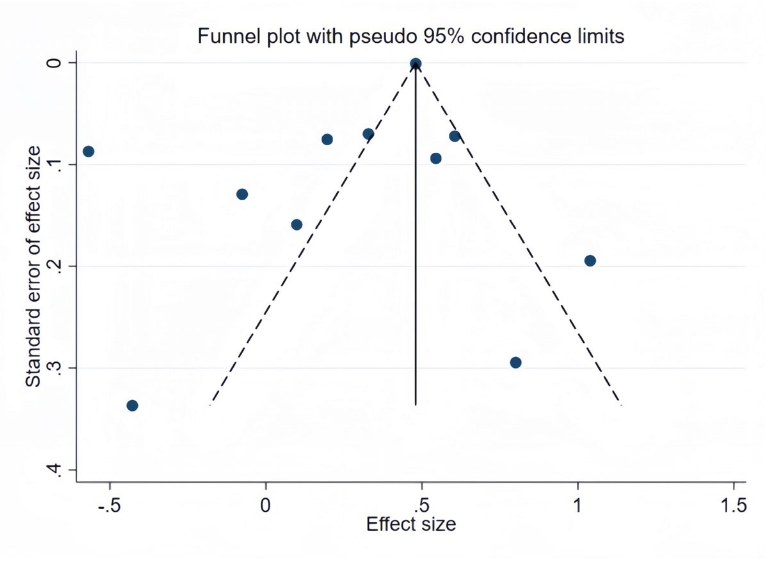

Supplementary Figure S7. Egger's regression test for the association between HL and the incidence of stroke and MI as a whole.

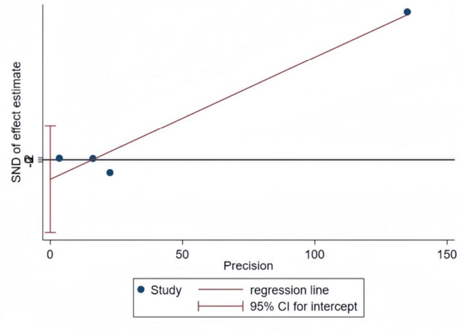


```
Egger's test for small-study effects:
Regress standard normal deviate of intervention
  effect estimate against its standard error

.
Number of studies =  4                                Root MSE      =   18.53
------------------------------------------------------------------------------
     Std_Eff |     Coef.   Std. Err.      t    P>|t|     [95% Conf. Interval]
-------------+----------------------------------------------------------------
       slope |  1.185767   .1754228     6.76   0.021     .4309839    1.940551
        bias | -18.89064   12.08682    -1.56   0.258    -70.89601    33.11474
------------------------------------------------------------------------------

Test of H0: no small-study effects          P = 0.258
```

Supplementary Figure S8. Forest plots of sensitivity meta-analyses synthesizing hazard ratios (HRs) only: (left) composite outcome of stroke or myocardial infarction (MI) (n=8 studies); (right) stroke outcome only (n=5 studies).

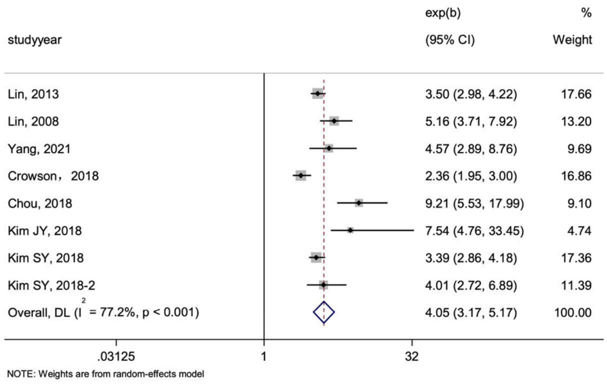

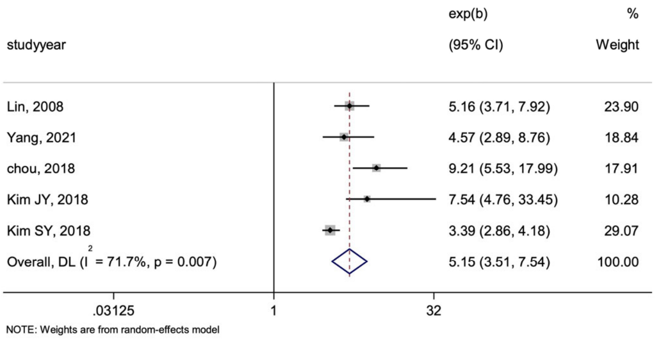

Supplement: Supplementary file 1 [file jcm-15-00577-s001.zip › jcm-4055297-supplementary.pdf]
